# The Noble Horse

**Published:** 1866-04

**Authors:** 


### THE NOBLE HORSE.

THE most loved and admired of all domestic animals deserves an additional mention in connection with the description of a model stable, as recommended by Mr. J. Wilkinson, Rural Architect and Landscape Gardener, of Baltimore, Maryland, and communicated to the *Maryland Farmer*, of that city, a monthly magazine, well deserving a large circulation. Mr. Wilkinson gives the pine plank floor the preference: "The worst of all is the clay floor. I lay the planks lengthwise, across the stalls, with a slope from front to rear. I lay the planks lengthwise across the stalls, and cut each plank under the partitions, and also in the centre of the stall. I lay them so as to leave an opening of half an inch between the ends, which forms a slot, or opening of a half inch in width and six feet in length, in the centre of the floor of each stall. I lay the floor so that it has a slope of three quarters of an inch from each side toward the centre, where the opening or slot is, but give the floor no obliquity 'fore and aft.' By this arrangement I accomplish a double object, namely, that of giving the animal the position he instinctively always seeks when in the pasture, by lying with the back 'up hill,' or the highest, for either side is higher than the centre.

"My mode of ventilation consists in making the building as close as practicable, with the exception of the ingress and egress openings for ventilation; the former I place in the floor, immediately in front of the horses; the latter on the highest part of the roof, having no obstructions between these two points. I take the air into the ground, if practicable, at the distance of one hundred feet from the stable, and lay an air-duct of proper dimensions from the receiving-well to an area under the feeding passage-floor, that portion of the floor over it being latticed.

"By this arrangement I take the air into the building, summer and winter, at the temperature of the ground, at the depth of which I lay the duct. Thus it is warmer than the external atmosphere in winter, and cooler in summer, and every breath is fresh and pure—a condition of things widely contrasting with that I have described, the result of the ordinary stable arrangement, which no one conversant with the subject will deny.

"I feed the hay directly from the hay-loft through a sheet-iron hay-tube, which is eighteen inches in diameter at the top, and twenty-four inches at the base. It stands on the tie-rail, level with the top of the manger, between two stalls, and extends to the level with the surface of the hay-loft floor. The top of it is covered. There are openings on either side, so that two animals eat from one tube. There is no waste of hay nor dust made in the stable in feeding it. The grain-feeding manger is of cast-iron, is hung on hinges, hence, may be removed and cleansed at pleasure.

"I also secure the most perfect drainage, by allowing the urine to fall directly through the slot in the floor, where it is received into the V-shaped iron gutter under the floor, which discharges it into a main gutter under the floor in the rear of the line of stalls, outside of the stable.

"By this arrangement neither the bedding nor the floor is wet, only where the urine falls, which is usually over the opening in the floor, hence, the bedding will be less saturated with urine, even if it is allowed to lie for a fortnight without moving it, than it will be in a single night with the use of the tight floor laid with a slope from front to rear. The urine usually falls about five feet from the rear of the stall, and if the floor has a slope to the rear, it will, in running that distance, be obstructed and spread over nearly the entire width of the stall before it is discharged into the surface-gutter in the rear of the stalls. In this gutter it is still more obstructed by the excrement; and the result is, that when the animal lies, he presses the bedding on to the floor, surcharged with putrescent urine, which it absorbs, and saturates the belly, thighs, and tail of the animal, and the blanket; all of which is avoided by my arrangement.

"This excessively filthy condition of the animal, revolting as it is to all who have proper appreciation of 'cleanliness, which is next to godliness,' is not the worst feature consequent upon this barbarous, though universal state of things. The heat of the bed and animal lying on the bed and floor, fully saturated with putrid urine, will give off the most fetid gases, which the animal must breathe over and over again during the whole night."

